# Cognition, internalizing symptoms and neonatal cingulate-amygdala interaction in children born very low birth weight

**DOI:** 10.3389/fped.2026.1880770

**Published:** 2026-09-04

**Authors:** Carmen Rodríguez-Barrios, Irene Gutiérrez-Rosa, Natalia Jiménez-Luque, Yolanda Marín-Almagro, Manuel Lubián-Gutiérrez, Bahram Jafrasteh, Simón Pedro Lubián-López, Isabel Benavente-Fernández

**Affiliations:** 1 Department of Paediatrics, Puerta del Mar University Hospital, Cadiz, Spain; 2 Biomedical Research and Innovation Institute of Cadiz (INiBICA), Research Unit, Puerta del Mar University Hospital, Cadiz, Spain; 3 Department of Child and Mother Health and Radiology, Medical School, University of Cadiz, Cadiz, Spain

**Keywords:** amygdala, cingulate cortex, cognitive outcome, internalizing symptoms, neonatal MRI, neurodevelopment, preterm birth

## Abstract

**Introduction:**

Children born with very low birth weight are at increased risk of cognitive difficulties and internalizing symptoms, including anxiety, withdrawal, and depressive features. However, the early neurodevelopmental pathways that connect neonatal brain maturation with emotional outcomes remain incompletely understood. This study examined whether internalizing symptoms at preschool age are associated with cognitive performance in children born with very low birth weight, and whether the interaction between cingulate and amygdala volumes is related to these symptoms directly or through cognition.

**Methodology:**

This longitudinal cohort included infants born with very low birth weight who were admitted to a neonatal intensive care unit between 2018 and 2021. Participants underwent brain magnetic resonance imaging at term-equivalent age and follow-up assessment at 4 to 6 years. Cognitive performance was evaluated with the Wechsler Preschool and Primary Scale of Intelligence, and internalizing symptoms were assessed with the Child Behavior Checklist. Adjusted linear regression models examined associations between cognition, neonatal regional brain volumes, and internalizing symptom scores. An observed-variable path model tested direct and indirect associations involving cingulate-amygdala interaction and cognition, and receiver operating characteristic analysis evaluated discrimination for clinically relevant internalizing symptoms.

**Results:**

Ninety-five children were included; mean gestational age was 29.4 ± 2.1 weeks and mean birth weight 1191.6 ± 350.8 g. Clinically relevant internalizing symptoms were present in 24/95 children (25.3%). Lower internalizing T-scores were associated with Higher IQ after adjustment (B = −0.282; *P* = 0.020). The cingulate-amygdala interaction was associated with internalizing symptoms (B = −60.377; *P* = 0.009). In path analysis, IQ was inversely associated with internalizing symptoms (B = −0.315; *P* < 0.001), and the indirect effect through IQ was significant (B = −19.449; *P* = 0.003). The MRI-based logistic model showed acceptable discrimination (area under the curve, 0.75).

**Discussion:**

Among children born VLBW, preschool internalizing symptoms were associated with poor cognitive performance and neonatal cingulate-amygdala interaction. These findings support interconnected brain-cognition-behavior pathways after preterm birth and underscore the value of integrated neurodevelopmental follow-up.

## 1 Introduction

Advances in perinatal care have substantially improved the survival of very low birthweight infants (VLBWI). Despite this progress, preterm (PT) birth remains strongly associated with adverse neurodevelopmental outcomes (1). By prematurely interrupting a critical phase of brain development, it exposes the immature brain to multiple insults, including white matter injury (WMI), germinal matrix-intraventricular hemorrhage (GMH-IVH), and impaired maturation. These processes may produce both overt structural injury and persistent disturbances in brain development, underlying a wide range of neurodevelopmental difficulties across infancy and childhood (2).

Internalizing problems, defined as emotional difficulties directed inward such as anxiety, depressive symptoms, and social withdrawal, are among the most common behavioral difficulties in children born PT (3). Beyond major neurological impairments, PT children more often show a characteristic preterm behavioral phenotype, marked by attentional, socioemotional, and especially internalizing difficulties (4). Consistent with this, studies have shown that children and adolescents born VLBWI are more vulnerable to anxiety, withdrawal, and depressive symptoms, with clinically relevant internalizing problems reported in approximately 15%–30% during early childhood (5–7).

Children born as VLBWI frequently show persistent cognitive difficulties, including lower intelligence scores and deficits in attention, working memory, processing speed, and cognitive flexibility (8, 9). These problems often continue into childhood and adolescence, affecting academic and functional outcomes and frequently co-occurring with behavioral difficulties, including internalizing symptoms (4, 10).

Early brain alterations may contribute to the cognitive and behavioral difficulties observed after PT birth (11–13). Among the brain regions potentially relevant to these outcomes, the amygdala and cingulate cortex represent complementary components of distributed systems involved in the processing of behaviorally salient information and the integration of affective and cognitive signals. The amygdala plays a central role in detecting and assigning significance to salient stimuli and in coordinating responses that influence learning and behavior (14), whereas the cingulate cortex contributes to the monitoring and integration of affective, cognitive, and behavioral information (15). These complementary functions, together with previous evidence linking amygdala- and cingulate-related measures to later internalizing and neurodevelopmental outcomes after PT birth, provided the theoretical basis for focusing on these regions in the present study.

However, the evidence concerning the amygdala and cingulate system derives from different neuroimaging modalities and developmental stages. Structurally, very PT infants have shown altered amygdala development, including smaller amygdala volumes at term-equivalent age (TEA) compared with term-born infants (16). Functionally, neonatal resting-state studies have shown that amygdala connectivity is reduced in magnitude in very PT infants and that neonatal connectivity between the amygdala and regions including the medial prefrontal cortex, posterior cingulate cortex, anterior insula, and dorsal anterior cingulate cortex at TEA is associated with internalizing symptoms at 2 years of age (17). Amygdala resting-state functional connectivity assessed at TEA has also been associated with later socio-emotional outcomes at 4–7 years (18). Importantly, these studies provide evidence concerning functional connectivity and should not be interpreted as demonstrating associations with regional volume.

Evidence involving the cingulate system is similarly modality-specific. Neonatal microstructural variation in the dorsal anterior cingulum bundle has been associated with later symptoms of the PT behavioral phenotype at 5 years of age; however, this finding concerns a white matter pathway rather than cingulate cortical volume (19). At school age, amygdala subnuclear volumes and amygdala network connectivity have also been associated with caregiver-reported social-emotional difficulties in children born very PT (20). More specifically, it has been reported smaller amygdala volumes at TEA in very PT infants than in term-born controls and found that, among the PT infants, larger amygdala volumes were associated with a more intense escape response during the Laboratory Temperament Assessment Battery fear episode at 12 months (16). In a separate cohort, Liverani et al. (21) examined cortical and deep gray matter, white matter, cerebellar, cerebrospinal fluid, and amygdala volumes at TEA and assessed behavioral outcomes at 5 years using the Strengths and Difficulties Questionnaire (SDQ). In their predictive analysis, white matter, amygdala, and cerebellar volumes were the most informative volumetric variables for the SDQ Emotional Symptoms score. These findings illustrate that associations between neonatal brain structure and later behavioral outcomes depend on both the brain measure examined and the specific behavioral construct assessed. Collectively, these findings support the relevance of distributed limbic-frontocingulate systems while leaving the specific association of neonatal cingulate and amygdala volumes, and their statistical interaction, incompletely understood.

The CBCL Internalizing Problems scale was selected as a standardized caregiver-reported measure of broad internalizing symptom burden (22). However, the CBCL does not directly assess emotion-regulation capacity or the functional integrity of a specific neural circuit. Accordingly, the present study examines whether neonatal regional volumetric variation is associated with later internalizing symptoms, rather than directly testing a mechanism of emotion regulation.

Therefore, the aims of the present study were: to examine the association between cognitive performance and internalizing symptoms at preschool age in children born very PT; to evaluate whether cingulate and amygdala volumes measured at TEA, and their statistical interaction, were associated with later internalizing symptoms; and to examine whether the association between the volumetric interaction and internalizing symptoms showed a statistical indirect association through cognitive performance. Because the regional volumetric model was identified through an exploratory model-selection procedure, the neuroimaging findings were considered hypothesis-generating.

## 2 Methodology

### 2.1 Study population

This study was a longitudinal cohort study of VLBWI admitted to the neonatal intensive care unit of Hospital Puerta del Mar between January 2018 and August 2021. Infants with a birth weight ≤1,500 g and/or GA ≤32 weeks at birth who were admitted to the neonatal intensive care unit (NICU). The exclusion criteria were the presence of congenital or genetic anomalies, metabolic disorders, and central nervous system infections. PT infants who died were also excluded. This study was approved by the Research and Ethics Committee, and all parents or guardians of the participants provided informed consent.

The variables analysed included prenatal variables (multiple pregnancy, *in vitro* fertilization, preeclampsia, gestational diabetes, chorioamnionitis, maternal education level, maternal age); perinatal variables (GA at birth, sex, birth weight, type of delivery); and postnatal variables (bronchopulmonary dysplasia (BPD), hemodynamically significant persistent ductus arteriosus (HSPDA), late sepsis, necrotizing enterocolitis, retinopathy of prematurity (ROP) and days of mechanical ventilation). The presence of neurological injury was assessed using magnetic resonance imaging (MRI) at TEA. For this purpose, we used the scale published by Kidokoro et al. (23), which separately grades the development and lesions of the cortical and deep gray matter, white matter, and cerebellum. The overall score obtained classifies the MRI findings as follows: normal (0–3 points) or abnormal: mild (4–7 points), moderate (8–11 points), and severe (≥12 points). Children with a score of less than 8 points were considered to have normal MRI findings or findings compatible with mild involvement, whereas children with a score equal to or greater than 8 points had findings suggestive of moderate or severe brain injury.

The GMH-IVH was graded according to the classification proposed by Volpe. Grades I and II GMH-IVH were considered mild, whereas grade III and PHI were considered moderate to severe GMH-IVH (24).

### 2.2 Brain magnetic resonance imaging

MRI was performed using a 1.5 T MagnetoM Symphony scanner (Siemens Health Care, Erlangen, Germany) located in the radiology unit. T1-weighted images were obtained using a three-dimensional spoiled gradient [repetition time 1660 (RT)/echo time 5.16 (ET)] and transverse T2-weighted images with turbo spin echo (4180.00/98.00).

Segmentation of regional brain volumes for the structures of interest was performed by multi-atlas segmentation using MELAGE, a software platform for segmentation, visualization, and semi-manual correction developed by our group (25, 26). Regional brain volumes were expressed in cm^3^. For bilateral structures, averaged regional volumes were used in the analyses.

The segmentation of the brain structures in T1-weighted images involves a systematic approach that enhances accuracy and precision. The process begins with brain extraction, using a specialized image reconstruction and brain extraction tool. Following extraction, brain tissue was segmented using an enhanced spatial fuzzy C-means algorithm for 3D T1 MRI segmentation (26). The resulting tissue segmentation maps, particularly those from the gray matter region, are then subsequently employed to register each T1 image to the MCRIB 2.0 atlas (27). This registration process aligns the individual images with a standardized anatomic framework, facilitating consistent comparisons across subjects. Once registration is complete, label information from the MCRIB 2.0 atlas is assigned to various regions of interest in the T1 images. This step is pivotal because it provides a comprehensive mapping of the anatomic structures within the images. The resultant segmentation was underwent visual quality control and specialist review within the MELAGE platform.

### 2.3 Neurodevelopment and behavioral assessment

Cognitive performance was assessed using the Wechsler Preschool and Primary Scale of Intelligence-IV Edition (WPPSI-IV) (28). The primary cognitive outcome was total Intelligence Quotient (IQ). As a secondary/sensitivity outcome, poor cognitive outcome was defined as total IQ less than 85. Cognitive assessments were administered by trained psychologists blinded to neonatal MRI findings.

Behavioral outcome was assessed using the Child Behavior Checklist (CBCL) (22). For the present analysis, we focused on the Internalizing Problems scale, using both the continuous T-score and a dichotomized clinically relevant outcome defined as a T-score >65.

### 2.4 Statistical analysis

Categorical variables were summarized as frequencies and percentages, whereas continuous variables were summarized as mean ± standard deviation (SD) or median [interquartile range, IQR], as appropriate according to their distribution. The main behavioral outcome was the Internalizing Problems score, analyzed both as a continuous T-score and as a dichotomized clinically relevant outcome, defined as a T-score >65.

Regression coefficients from linear and logistic regression models are reported as unstandardized coefficients (B) with 95% confidence intervals (95% CI). For the path model, both unstandardized and standardized coefficients are reported to facilitate interpretation and comparison across paths.

The association between IQ score and internalizing problems T-score was assessed using linear regression. Initial models were adjusted for GA at birth and chronological age at assessment. Fully adjusted models additionally included prespecified perinatal and neonatal covariates.

Based on the study hypothesis and previous evidence implicating limbic-frontocingulate systems in behavioral outcomes after preterm birth, the primary MRI analysis focused on cingulate volume, amygdala volume, and their statistical interaction. Before calculating the interaction term, continuous cingulate and amygdala volumes were mean-centered. Linear regression models were fitted to examine their association with the CBCL Internalizing Problems T-score, adjusting for gestational age at birth and postmenstrual age at term-equivalent MRI. Additional regional volumetric interaction models involving other neonatal brain structures were considered as secondary sensitivity analyses and are reported in the Supplementary Material.

For all linear regression models, linearity and homoscedasticity were assessed using residual-versus-fitted plots and Cook-Weisberg test. The distribution of residuals was examined using quantile–quantile plots. Multicollinearity was assessed using variance inflation factors. Potentially influential observations were evaluated using studentized residuals, leverage values, and Cook's distance. Sensitivity analyses were performed after excluding observations meeting prespecified criteria for substantial influence. For each linear regression model, the analytical sample size, overall F statistic, model *P* value, R^2^, and adjusted R^2^ were reported.

The cingulate-amygdala interaction term was subsequently entered into an observed-variable path model to estimate direct, indirect, and total associations involving cognitive performance and Internalizing Problems T-scores. Direct, indirect, and total effects were reported as unstandardized and standardized coefficients with 95% CI. The indirect effect was estimated using 5,000 bootstrap replications. Model identification was assessed before evaluating global fit.

For the dichotomized internalizing outcome, a logistic regression model was fitted including gestational age at birth, postmenstrual age at term-equivalent MRI, cingulate volume, amygdala volume, and their interaction. Results are reported as odds ratios with 95% confidence intervals. Model discrimination was quantified using the area under the receiver operating characteristic curve with its 95% confidence interval. The probability threshold maximizing the Youden index was identified, and the corresponding sensitivity and specificity were calculated. Apparent calibration was evaluated using the calibration intercept, calibration slope, and Brier score.

Statistical analysis was performed using Stata 16.0 (Stata Statistical Software: Release 16. College Station, TX: StataCorpLP). Statistical significance was defined as a two-sided *p* value <0.05.

## 3 Results

### 3.1 Study population

**Figure 1:**
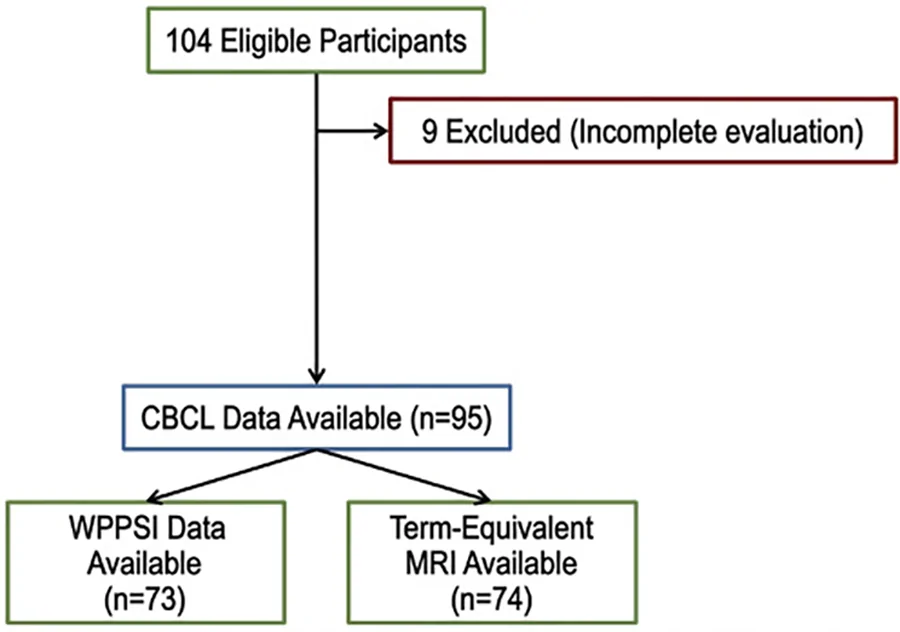
Flow diagram of the study population*.* CBCL, Child Behavior Checklist; WPPSI, Wechsler Preschool and Primary Scale of Intelligence; MRI, magnetic resonance imaging.

Among 104 children born VP who were eligible for follow-up assessment at 4–6 years of age, 9 did not complete the full assessment, therefore, 95 were included in the final study sample. Mean GA at birth was 29.4 ± 2.1 weeks, 49 (52.7%) were female, and mean birth weight was 1191.6 ± 350.8 g. Among infants with available neuroimaging data moderate-to-severe MRI abnormalities based on the Kidokoro score were identified in 9 patients, and grade 3 GMH-IVH and/or PHI was present in 7 patients (The other variables are described in detail in Table 1, and the study flowchart is presented in Figure 1).

**Table 1:** Perinatal characteristics of the patients who underwent assessment using the CBCL. Continuous variables are presented as mean ± SD or median [IQR], and categorical variables as n/N (%).

Characteristic		Category/available *N*	Value
Overall sample		*N* = 95	
GA at birth (weeks)		29.427 (±2.169)	
Sex (F)		49 (52.69%)	
Birth weight (g)		1191.559 (±350.749)	
SGA		*N* = 93	13 (13.98%)
Maternal age at delivery (years)		*N* = 93	33.193 (±5.724)
Chronological age at assessment (years)		4.753 (±0.591)	
Caesarean section		60 (63.16%)	
Prenatal steroids		*N* = 94	78 (82.98%)
Chorioamnionitis		*N* = 77	21 (27.27%)
Multiple pregnancy		*N* = 93	32 (34.41%)
IVF		*N* = 93	15 (16.13%)
Preeclampsia		*N* = 86	17 (19.77%)
Diabetes		8 (8.51%)	
Mothers’ level of education (*N* = 92)	Basic	69 (75%)	
	Intermediate	19 (20.65%)	
	Advanced	4 (4.35%)	
BPD (mod-severe)		*N* = 72	11 (14.86%)
HSPDA		*N* = 72	8 (11.11%)
Late onset sepsis		16 (16.84%)	
Severe NEC		1 (1.05%)	
ROP (mod-severe)		*N* = 71	3 (4.23%)
Brain injury as Kidokoro score (mod-severe)		*N* = 69	9 (13.04%)
GMH-IVH	No	53 (71.62%)	
	grade 1	10 (13.51%)	
	grade 2	4 (5.41%)	
	grade 3	4 (5.41%)	
	PHI	3 (4.05%)	
GMH 3 and/or PHI		*N* = 70	7 (10%)

GA, gestational age; SGA, small for gestational age; IVF, *in vitro* fertilization; BPD, bronchopulmonary dysplasia; HSPDA, hemodynamic significant patent ductus arteriosus with hemodynamic significance; NEC, necrotizing enterocolitis; ROP, retinopathy of prematurity; GMH-IVH, germinal matrix hemorrhage-intraventricular hemorrhage; PHI, periventricular hemorrhagic infarction; MV, mechanical ventilation.

Clinically relevant CBCL scores for internalizing problems were identified in 24/95 (25.3%). Mean internalizing score was 58.400 ± 11.348. WPPSI assessments were available in 73 children, of whom 13 had a score below 85, consistent with poor cognitive outcome.

### 3.2 Associations of internalizing problems with cognitive outcomes

In adjusted linear regression models including GA at birth and chronological age at assessment, higher IQ scores were associated with lower internalizing T-scores. (B = −0.396; 95% CI −0.691 to −0.100; *p* = 0.010). In the fully adjusted model, birth weight, BPD, late-onset sepsis, Kidokoro moderate-to-severe brain injury, and WMI were additionally included, as these perinatal variables were considered the most clinically relevant and were significantly associated with the outcome in bivariate analyses. The association remained significant (B = −0.282; *p* = 0.020) (Table 2), supporting a consistent relationship between poorer cognitive outcome and greater internalizing symptom burden.

**Table 2:** Fully adjusted model of internalizing problems including cognitive outcome (WPPSI) and perinatal covariates.

Predictor	B coefficient	95% CI	*p* value
*IQ*	**−0**.**282**	**−0.519 to −0.045**	**0**.**020**
GA at birth	−1.041	−3.385 to 1.303	0.384
Chronological age at assessment	−0.090	−0.482 to 0.302	0.654
*Birth weight*	**0**.**017**	**0.003 to 0.032**	**0**.**019**
*BPD*	**14**.**491**	**5.505 to 23.477**	**0**.**002**
Late-onset sepsis, yes	3.822	−5.938 to 13.582	0.443
Kidokoro moderate-severe, yes	−1.470	−8.794 to 5.854	0.694
*WMI*	**−12**.**633**	**−19.915 to −5.351**	**<0**.**001**
*N* = 71 R^2^ = 0.332. Adjusted R^2^ = 0.238. F(8,57) = 3.53. *P* = 0.002.			

WPPSI, Wechsler Preschool and Primary Scale of Intelligence; GA, Gestational Age; BPD, Bronchopulmonary dysplasia; WMI, white matter injury.

Bold values indicate statistically significant associations (*p* < 0.05).

### 3.3 Selection of term-equivalent MRI volumetric interaction models associated with internalizing problems

**Figure 2:**
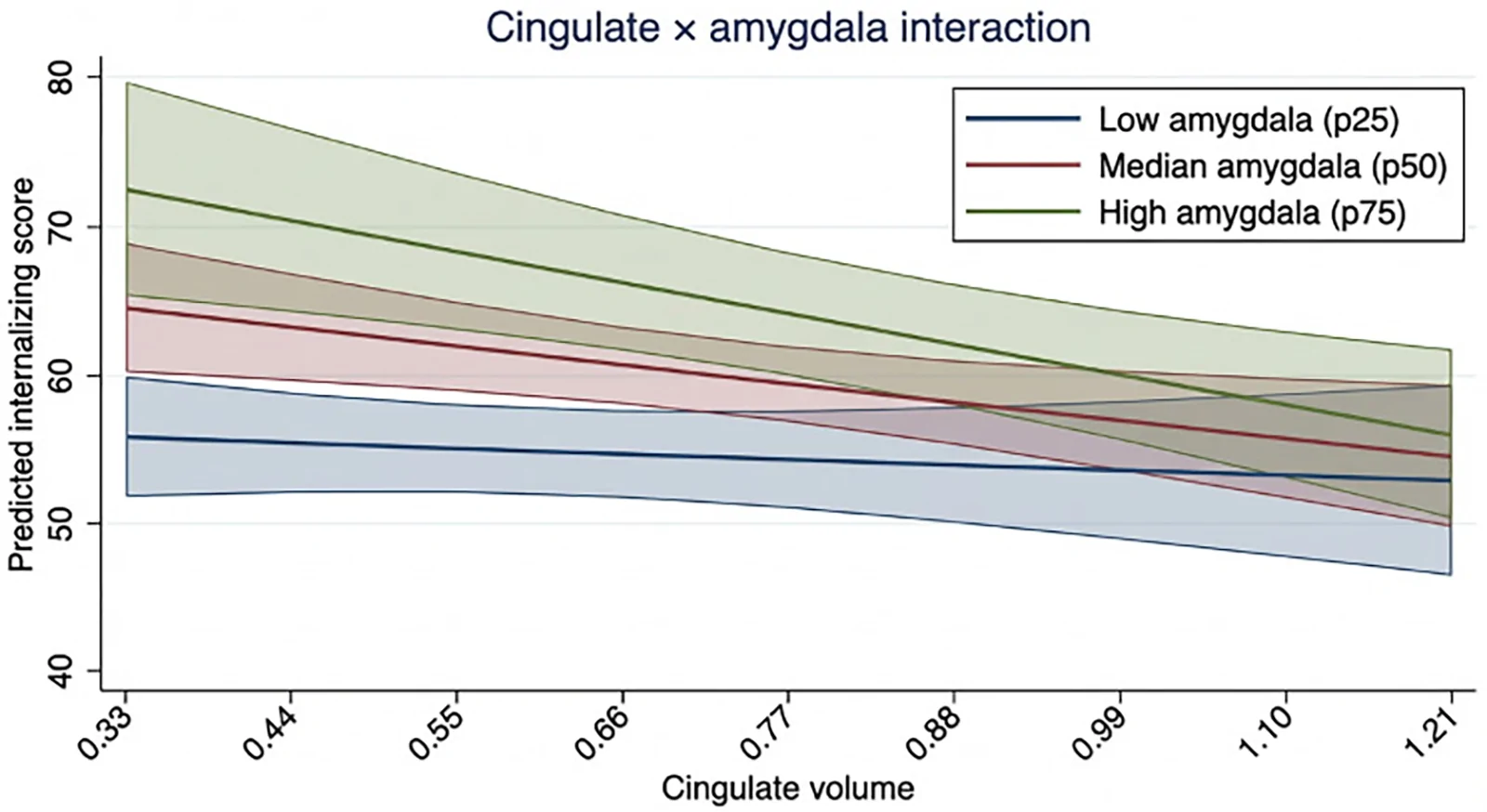
Predicted internalizing score according to cingulate volume at low (p25), median (p50), and high (p75) amygdala volumes.

Among the 10 best-fitting models identified by the all-subsets approach (Supplementary Material Table A1), the model including cingulate volume, amygdala volume, and their interaction provided the best overall fit. In this model, the cingulate-amygdala interaction was significantly associated with internalizing problems (B = −60.377; *p* = 0.009). Amygdala volume was also positively associated with internalizing T-score (B = 85.715, *p* < 0.001), whereas cingulate volume showed no association (B = 24.790, *p* = 0.064) (Table 3 and Figure 2). Higher amygdala volume was associated with higher predicted internalizing scores, particularly at lower cingulate volumes. In contrast, as cingulate volume increased, the difference in predicted internalizing scores across amygdala levels narrowed, consistent with a negative cingulate-amygdala interaction. Thus, the association between amygdala volume and internalizing symptoms appeared to be attenuated at higher cingulate volumes.

**Table 3:** Linear regression model including cingulate volume, amygdala volume, and their interaction.

Variable	B coefficient	95% CI	*p*-value
Cingulate volume	24.790	−1.472 to 51.052	0.064
*Amygdala volume*	**85**.**715**	**40.884 to 130.546**	**<0**.**001**
*Cingulate × Amygdala*	**−60**.**377**	**−105.135 to −15.618**	**0**.**009**
*N* = 72 R^2^/Adj. R^2^= 0.249/0.192 F statistic = 4.38 Model *p*-value = 0.0017			

The model was adjusted for GA at birth and PMA at MRI; these coefficients, together with the constant, are not shown in the main table for brevity. Adjustment coefficients were: GA at birth, B = 0.425, 95% CI −0.740 to 1.591, *p* = 0.469; PMA at MRI, B = −0.224, 95% CI −0.801 to 0.353, *p* = 0.440; constant, B = 13.270, 95% CI −26.588 to 53.128, *p* = 0.509. GA, gestational age; PMA, postmenstrual age.

Bold values indicate statistically significant associations (*p* < 0.05).

### 3.4 Path model linking internalizing symptoms, cingulate-amygdala interaction, and cognitive outcome

**Figure 3:**
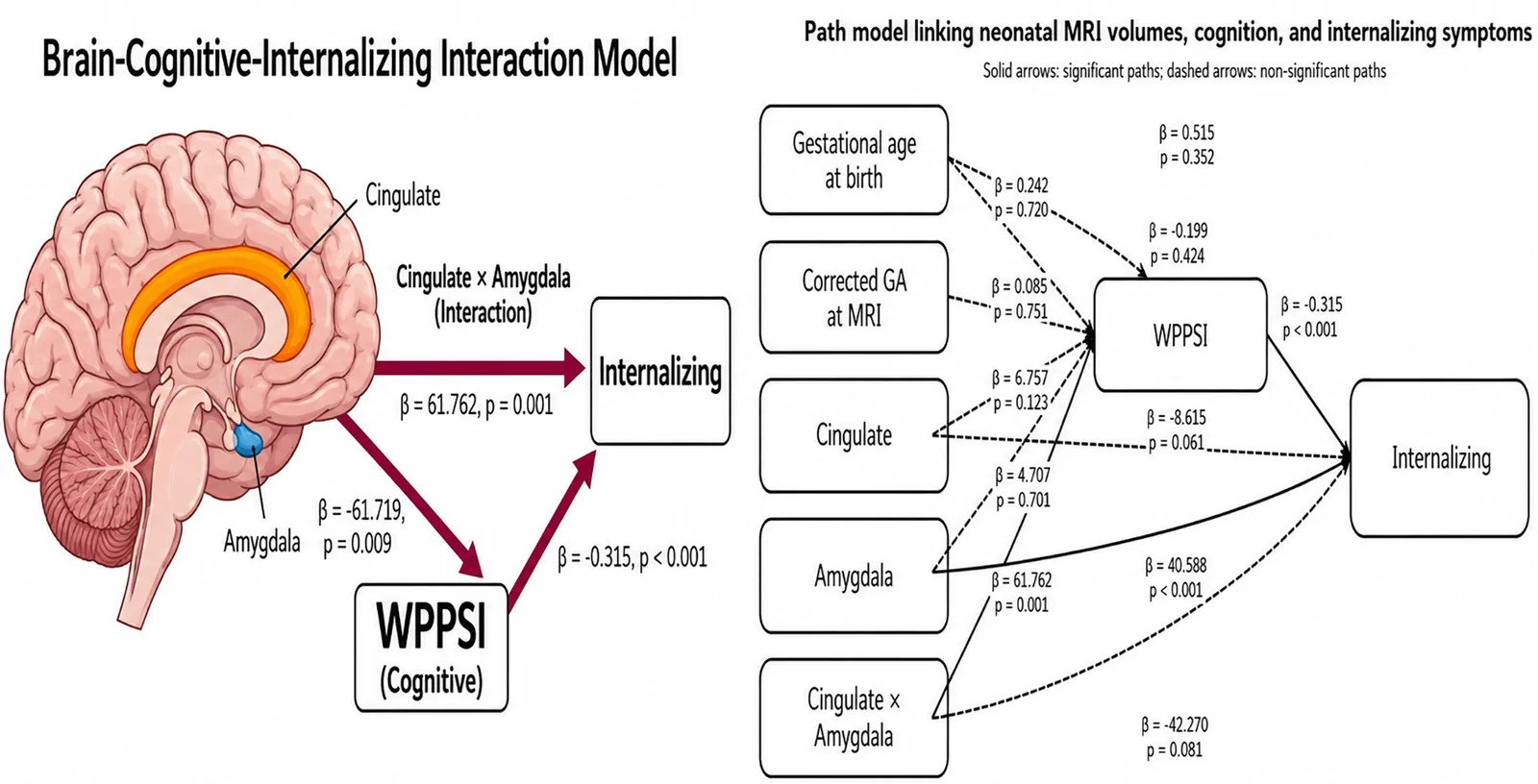
Observed-variable path model examining the direct relationships between gestational age at birth, corrected age at term-equivalent MRI, cingulate volume, amygdala volume, their interaction, cognitive outcome (WPPSI), and internalizing symptoms.

Given the observed association between internalizing problems and cognitive outcome, a path model was used to examine the relationships among GA at birth, PMA at MRI, cingulate volume, amygdala volume, their interaction, IQ score, and internalizing symptons within a single analytical framework (Supplementary Tables A2–A5). In this model, higher WPPSI scores were associated with lower internalizing scores (B = −0.315, *p* < 0.001). The cingulate and amygdala interaction was significantly associated with WPPSI (B = 61.762, *p* = 0.001), whereas its direct association with internalizing symptoms was attenuated and no longer statistically significant after inclusion of cognition (B = −42.270, *p* = 0.081) (Figure 3). The indirect effect through WPPSI was significant (B = −19.449, *p* = 0.003), and the total effect of the cingulate and amygdala interaction on internalizing symptoms remained significant (B = −61.719, *p* = 0.009). These findings support an indirect pathway linking the cingulate-amygdala interaction to internalizing symptoms through cognitive outcome. (Table 4).

**Table 4:** Summary of the SEM model linking neonatal MRI volumes, WPPSI, and internalizing symptoms**.**

Effect type	Path	B coefficient	95% CI	*p*-value
Direct path	Cingulate × Amygdala → WPPSI	61.762	23.896 to 99.627	0.001
Direct path	WPPSI → Internalizing	−0.315	−0.453 to −0.177	<0.001
Direct path	Cingulate × Amygdala → Internalizing	−42.270	−91.900 to 7.360	0.081
*Indirect effect*	Cingulate × Amygdala → WPPSI → Internalizing	−19.449	−37.697 to −5.635	0.003
*Total effect*	Cingulate × Amygdala → Internalizing	−61.719	−113.305 to −11.518	0.009

WPPSI, Wechsler Preschool and Primary Scale of Intelligence.

**Figure 4:**
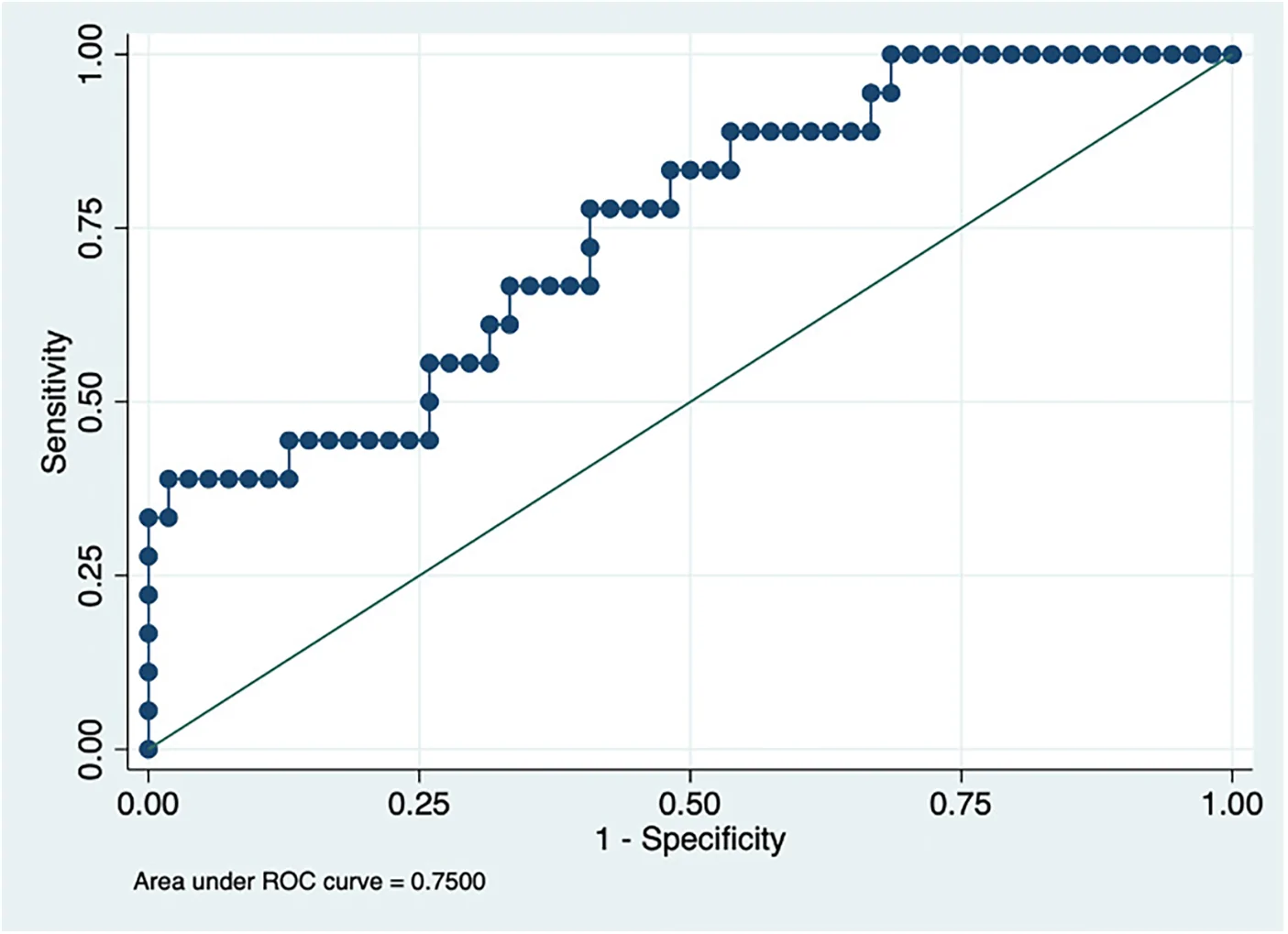
Receiver operating characteristic (ROC) curve for the logistic model predicting abnormal internalizing status from gestational age at birth, postmenstrual age at term-equivalent MRI, cingulate volume, amygdala volume, and their interaction.

To explore the discriminative performance of this MRI model for clinically relevant internalizing status, a logistic regression model including GA, PMA at MRI, cingulate volume, amygdala volume, and their interaction was evaluated with ROC analysis. The area under the ROC curve was 0.75 (Figure 4), indicating acceptable discrimination, corresponding to a sensitivity of 77.8% and a specificity of 59.3% (Table 5). Thus, the model showed moderate ability to classify children with abnormal internalizing scores.

**Table 5:** Receiver operating characteristic (ROC) performance of the logistic model predicting abnormal internalizing status from gestational age at birth, postmenstrual age at term-equivalent MRI, cingulate volume, amygdala volume, and their interaction.

Outcome	AUC	95% CI	Sensitivity	Specificity
Internalizing	0.750	0.609–0.867	0.778	0.593

AUC, area under the curve.

## 4 Discussion

In this cohort of children born very PT, internalizing symptoms at preschool age were common, with clinically relevant scores identified in 24 of 95 children (25.3%). Two main findings emerged from the present study. First, poor cognitive performance was associated with greater internalizing symptom burden, even after adjustment for relevant perinatal and neonatal factors. Second, in an exploratory model-selection analysis, the model containing cingulate volume, amygdala volume, and their statistical interaction showed the best fit among the candidate regional volumetric models. This finding extends previous work based primarily on neonatal amygdala functional connectivity, cingulum-bundle microstructure, or individual regional volumes by examining whether the relationship between two neonatal regional volumes was associated with later internalizing symptom burden. Given the data-driven model-selection procedure and the modest sample size, this result should be regarded as hypothesis-generating and requires independent replication.

The frequency of clinically relevant internalizing difficulties observed in our cohort is consistent with previous descriptions of the behavioral phenotype associated with PT birth, in which emotional symptoms, are more prominent than overt disruptive behavior. Rather than representing isolated difficulties, these symptoms are increasingly understood as part of a broader neurodevelopmental profile following preterm birth (4, 29).

Behavioral symptoms were also closely related to neurodevelopmental performance. The association between lower WPPSI scores and higher internalizing symptoms is clinically relevant. Cognitive difficulties are among the most consistent long-term sequelae of prematurity, but their relationship with emotional symptoms is less often examined within an integrated framework (30). The inverse association between WPPSI-IV IQ and CBCL Internalizing Problems T-scores observed in our cohort is consistent with previous evidence that cognitive and behavioral difficulties frequently co-occur after very PT birth. Previous studies have similarly shown that poorer executive functioning is associated with greater behavioral symptom burden in children born very PT (31). Therefore, the association between cognition and internalizing symptoms should be regarded as a confirmatory rather than a novel finding of the present study. Its relevance here lies in demonstrating this association within the same longitudinal cohort in which neonatal regional brain volumes were available, thereby allowing cognition, neonatal MRI findings, and later internalizing symptoms to be examined within a common analytical framework.

The TEA-MRI analysis provided a more specific pattern of associations with later CBCL Internalizing Problems T-scores. In the selected volumetric model, greater amygdala volume was associated with higher Internalizing Problems T-scores (B = 85.715, *p* < 0.001), whereas cingulate volume alone was not significantly associated with the outcome (B = 24.790, *p* = 0.064). Importantly, the cingulate-amygdala interaction was negative (B = −60.377, *p* = 0.009). As illustrated by the predicted values, the positive association between amygdala volume and Internalizing Problems T-scores was more pronounced at lower cingulate volumes and became progressively attenuated as cingulate volume increased. Thus, the principal MRI finding was not an isolated association of either region with later symptoms, but rather that the association between neonatal amygdala volume and later internalizing symptom burden varied according to cingulate volume. The amygdala and cingulate cortex participate in distributed systems relevant to salience processing, affective monitoring, and the integration of cognitive and emotional information (17, 18). However, the behavioral outcome examined in the present study was the CBCL Internalizing Problems T-score, a caregiver-reported measure of broad internalizing symptom burden. Accordingly, the neuroimaging findings should be interpreted specifically in relation to later parent-reported internalizing symptoms rather than as evidence for a particular psychological mechanism. Moreover, the statistical interaction between cingulate and amygdala volumes does not represent anatomical or functional connectivity between these regions.

In the path model, the association between the cingulate-amygdala interaction and internalizing symptoms was attenuated after inclusion of cognition, whereas the indirect effect through WPPSI remained significant. These findings suggest that cognitive performance may partly mediate the association between early limbic-cingulate interaction and later internalizing symptoms, consistent with a network-based model of PT neurodevelopment in which coordinated alterations in early brain maturation contribute to later behavioral outcomes (17, 19, 31, 32). The acceptable discrimination shown by the final model further supports the relevance of early amygdala-cingulate development in the neurodevelopmental pathways associated with later internalizing symptons.

Several limitations should be acknowledged. First, the sample size was modest, particularly for the MRI-based and path analyses, and only a subset of the full cohort had complete neuroimaging and cognitive data. Second, behavioral outcomes were based on parent-reported CBCL scores and therefore reflect symptom burden rather than formal psychiatric diagnoses. Although maternal education was recorded, comprehensive measures of the child's social and family environment were not available. Household income and deprivation, parental anxiety or depressive symptoms, parenting stress, family functioning, the home learning environment, adverse childhood experiences, and access to early developmental, psychological, or educational support may act as risk or protective factors and may confound or modify the associations between neonatal brain development and later outcomes. Third, although the analyses accounted for key maturational and neonatal variables, residual confounding related to family environment, parental mental health, and later developmental exposures cannot be excluded. Fourth, the MRI analyses were based on neonatal volumetric measures at TEA and do not capture later developmental trajectories, microstructural alterations, or functional connectivity more directly. Finally, because the final volumetric model emerged from a broader model-selection strategy, replication in independent cohorts will be necessary before firm conclusions can be drawn.

The present study has several strengths, including its longitudinal design from term-equivalent neonatal MRI to preschool follow-up, the combined assessment of brain structure, cognition, and behavior within the same cohort, and the use of both continuous and clinically relevant categorical internalizing outcomes. In addition, the analytical strategy integrated adjusted regression models, volumetric interaction analyses, and path modeling, allowing the association between early brain organization and later internalizing symptoms to be examined within a broader neurodevelopmental framework.

In summary, in children born very PT, internalizing symptoms at preschool age were associated with poorer cognitive performance and with early variation in neonatal cingulate-amygdala interaction. The relationship between this MRI pattern and later internalizing symptoms showed a significant statistical indirect association through cognition, supporting the view that internalizing symptoms after PT birth may emerge through interconnected brain-behavior developmental pathways. These findings reinforce the importance of considering cognition and internalizing problems together in follow-up programs for children born PT and suggest that early brain alterations in limbic-regulatory networks may contribute to later internalizing symptom burden.

## Data Availability

The original contributions presented in the study are included in the article/Supplementary Material, further inquiries can be directed to the corresponding author.
