# Supplementary material for: Cognition, internalizing symptoms and neonatal cingulate-amygdala interaction in children born very low birth weight
Source: Front Pediatr. 2026 Sep 4;14:1880770. doi: 10.3389/fped.2026.1880770 (PMC13585949; doi:10.3389/fped.2026.1880770)
Supplement: Supplementary file 1 [file Table1.docx]

Supplementary Material

**Supplementary Table A1. Top 10 candidate interaction models for the internalizing scale**

| **Rank** | **Interaction term** | **N** | **R²** | **Adjusted R²** | **Interaction β** | **p-value** |
| --- | --- | --- | --- | --- | --- | --- |
| 1 | GA+PMA+Cingulate × Amygdala | 72 | 0.249 | 0.192 | -60.377 | 0.009 |
| 2 | GA+PMA+Putamen × Amygdala | 72 | 0.245 | 0.188 | -40.418 | 0.061 |
| 3 | GA+PMA+Gray matter × Amygdala | 72 | 0.238 | 0.180 | -0.720 | 0.066 |
| 4 | GA+PMA+Thalamus × Cingulate | 72 | 0.223 | 0.165 | -13.531 | 0.001 |
| 5 | GA+PMA+Gray matter × White matter | 72 | 0.215 | 0.155 | -0.008 | 0.002 |
| 6 | GA+PMA+Thalamus × Amygdala | 72 | 0.197 | 0.136 | -26.063 | 0.017 |
| 7 | GA+PMA+Putamen × Cingulate | 72 | 0.194 | 0.133 | -30.126 | 0.003 |
| 8 | GA+PMA+Hippocampus × Cingulate | 72 | 0.187 | 0.125 | -25.293 | 0.013 |
| 9 | GA+PMA+White matter × Amygdala | 72 | 0.183 | 0.121 | -11.412 | 0.024 |
| 10 | GA+PMA+Hippocampus × Amygdala | 72 | 0.183 | 0.121 | -59.196 | 0.024 |

*GA: Gestational age, PMA: postmenstrual age.*

**SEM Path Model: Neonatal MRI Volumes, WPPSI, and Internalizing Symptoms**

**Supplementary Table A2. Model fit summary**

| **Model summary** | **Value** |
| --- | --- |
| Number of observations | 71 |
| Estimation method | ML with robust standard errors |
| Log pseudolikelihood | -775.725 |
| SRMR | 0.000 |
| Coefficient of determination (CD) | 0.328 |
| Main significant path | WPPSI → Internalizing (β = -0.315, p < 0.001) |
| Significant indirect path | Cingulate × Amygdala → WPPSI → Internalizing (β = -19.449, p = 0.003) |

**Supplementary Table A3. Direct effects**

| **Path** | **Estimate / SE / p** | **95% CI** |
| --- | --- | --- |
| GA → WPPSI | β 0.242 SE 0.673 p 0.720 | -1.078 to 1.561 |
| PMA→ WPPSI | β 0.085 SE 0.266 p 0.751 | -0.437 to 0.606 |
| Cingulate → WPPSI | β 6.757 SE 4.381 p 0.123 | -1.828 to 15.343 |
| Amygdala → WPPSI | β 4.707 SE 12.261 p 0.701 | -19.324 to 28.738 |
| Cingulate × Amygdala → WPPSI | β 61.762 SE 18.616 p 0.001 | 25.274 to 98.249 |
| WPPSI → Internalizing | β -0.315 SE 0.067 p <0.001 | -0.447 to -0.183 |
| GA → Internalizing | β 0.515 SE 0.553 p 0.352 | -0.569 to 1.598 |
| PMA → Internalizing | β -0.199 SE 0.249 p 0.424 | -0.688 to 0.289 |
| Cingulate → Internalizing | β -8.615 SE 4.604 p 0.061 | -17.639 to 0.409 |
| Amygdala → Internalizing | β 40.588 SE 11.176 p <0.001 | 18.683 to 62.492 |
| Cingulate × Amygdala → Internalizing | β -42.270 SE 24.212 p 0.081 | -89.725 to 5.185 |

**Supplementary Table A4. Indirect effects**

| **Indirect path to Internalizing via WPPSI** | **Estimate / SE / p** | **95% CI** |
| --- | --- | --- |
| GA → WPPSI → Internalizing | β -0.076 SE 0.206 p 0.711 | -0.479 to 0.327 |
| PMA → WPPSI → Internalizing | β -0.027 SE 0.085 p 0.754 | -0.193 to 0.140 |
| Cingulate → WPPSI → Internalizing | β -2.128 SE 1.521 p 0.162 | -5.109 to 0.853 |
| Amygdala → WPPSI → Internalizing | β -1.482 SE 3.784 p 0.695 | -8.899 to 5.935 |
| Cingulate × Amygdala → WPPSI → Internalizing | β -19.449 SE 6.561 p 0.003 | -32.307 to -6.590 |

**Supplementary Table A5. Total effects on internalizing symptoms**

| **Total effect on Internalizing** | **Estimate / SE / p** | **95% CI** |
| --- | --- | --- |
| GA | β 0.438 SE 0.607 p 0.470 | -0.751 to 1.628 |
| PMA | β -0.226 SE 0.245 p 0.356 | -0.705 to 0.253 |
| Cingulate | β -10.743 SE 4.497 p 0.017 | -19.558 to -1.928 |
| Amygdala | β 39.105 SE 10.893 p <0.001 | 17.755 to 60.456 |
| Cingulate × Amygdala | β -61.719 SE 23.707 p 0.009 | -108.184 to -15.253 |

WPPSI, Wechsler Preschool and Primary Scale of Intelligence; SRMR, standardized root mean squared residual; CD, coefficient of determination.
